# 776 Importance of Adequate Nutrition Support in Severely Burned Patients

**DOI:** 10.1093/jbcr/irad045.251

**Published:** 2023-05-15

**Authors:** Amanda Stone

**Affiliations:** Lehigh Valley Health Network- Cedar Crest Campus, Doylestown, Pennsylvania

## Abstract

**Introduction:**

The importance of optimal nutrition for burned patients has been well established through research. Provision of adequate nutrition is believed to improve healing outcomes, reduce complications and overall length of stay for burned patients. Enteral Nutrition (EN) support is the favored method of feeding for a severely burned patient, however timing of initiation of feeding and ability to meet estimated nutritional needs throughout a hospital stay still needs to be further established. The objective of this study was to evaluate not only the timing of initiation of EN for severely burned patients, but also the adequacy of the nutritional provisions the patients received.

**Methods:**

Patients admitted to a Regional Burn Center with a burn ≥20% TBSA, intubated and started on EN support were eligible for the inclusion in this retrospective review. Specific variables that were assessed included time for burn team to consult Registered Dietitian (RD), time from consult until RD assessed patient, time from admission to initiation per order, total tube feed volume (in mL) actually infused per RN documentation, and percentage of EN received compared to order.

**Results:**

Forty-two patients were included in this retrospective review. All patients had EN initiated within 24 hours of admission. Out of twenty-five patients that specific EN initiation time (by the minute) was able to be identified, the average time for EN to start was 9.5 hours. Average time for enteral nutrition to reach RD recommended goal rate was 26.2 hours, with 59% of patients reaching goal rate in ≤24 hours. Average amount of EN formula (in mL) patients actually received compared to what was ordered was 80.9%. Some complications observed for those patients whose EN did not reach goal rate in ≤24 hours or those who received a lower percentage of EN formula included difficulties with GI tolerance, high residuals, and incidence of ileus. However, these complications were only seen in 19% of patients included in the study.

**Conclusions:**

The average time for initiation of EN support was within the SCCM/ASPEN guidelines for the critically ill patient. Additionally, more than half the patients reached the RD recommended EN goal rate in ≤24 hours. The investigator found that early initiation of EN and aggressive advancement to goal, as tolerated, to be safe.

**Applicability of Research to Practice:**

A standard of practice for timing of EN initiation and advancing to goal would be beneficial, with possibility of reaching EN goal rates faster helping to increase the provisions of total EN received during admission and therefore closer meeting estimated nutritional needs. The effects of adequate nutrition in relation to overall burn healing / graft acceptance, complications, length of stay and mortality all warrant further studies prior to implementation of early EN/aggressive advancement to goal into practice.

